# Robot-Assisted Placement of Thoracic Carbon-Fiber-Reinforced Polyetheretherketone (CFR-Peek) Pedicle Screws in the Cervical Spine for Giant Cell Tumor: Technical Note

**DOI:** 10.3390/bioengineering13030361

**Published:** 2026-03-19

**Authors:** Emanuele Stucchi, Mario De Robertis, Gabriele Capo, Ali Baram, Giuseppe De Gennaro Aquino, Donato Creatura, Leonardo Anselmi, Maurizio Fornari, Federico Pessina, Carlo Brembilla

**Affiliations:** 1 Department of Neurosurgery, IRCCS Humanitas Research Hospital, Via Alessandro Manzoni 56, Rozzano, 20089 Milan, Italygiuseppe.degennaroaquino@humanitas.it (G.D.G.A.);; 2 Department of Biomedical Sciences, Humanitas University, Via Rita Levi Montalcini 4, Pieve Emanuele, 20090 Milan, Italy

**Keywords:** giant cell tumor of bone, Polyetheretherketone, robotic surgical procedures, cervical vertebrae, pedicle screws, spinal neoplasms

## Abstract

Carbon-Fiber-Reinforced Polyetheretherketone (CFR-PEEK) instrumentation is increasingly preferred in spinal oncology for its physical properties, minimizing imaging artifacts and facilitating precise postoperative radiotherapy planning and tumor surveillance. However, a significant technical limitation exists: the current unavailability of dedicated CFR-PEEK pedicle screws for the cervical spine. The smallest available implants are designed for thoracic use (minimum diameter 4.5 mm, minimum length 25 mm), posing substantial risks of neurovascular injury when applied to smaller cervical pedicles. We present a technical note/feasibility report illustrated by a single case of robot-assisted placement of thoracic CFR-PEEK screws in the cervical spine for the treatment of a C7 Giant Cell Tumor. Following neoadjuvant therapy with Denosumab, a single-stage, two-step circumferential resection and reconstruction was performed. The anterior step was complicated by an iatrogenic injury to the highly adherent left vertebral artery (VA), which was successfully repaired. Consequently, the posterior step required maximal precision to preserve the sole remaining intact VA on the right side. Given the anatomical mismatch between the 4.5 mm thoracic screws and the narrow cervical pedicles (measuring as narrow as 3.2 mm on the critical right side), robotic navigation (ExcelsiusGPS^®^) was utilized to plan and execute safe trajectories. Specifically, on the side of the intact VA, a small, controlled medial cortical violation was planned to avoid lateral vascular compromise. The procedure resulted in rigid, artifact-free stabilization with no immediate neurological sequelae. This single-case experience suggests that robotic guidance may facilitate adaptation of thoracic CFR-PEEK instrumentation to the cervical spine in selected oncologic scenarios; reproducibility, costs, and long-term outcomes remain uncertain.

## 1. Introduction

Giant Cell Tumor of Bone (GCTB) of the spine is a locally aggressive neoplasm that presents significant management challenges due to its high recurrence rate and proximity to critical neurovascular structures [1,2,3]. While en bloc resection (R0 margins) remains the gold standard for optimizing local control, a multimodal strategy involving neoadjuvant Denosumab followed by intralesional piecemeal resection is often a valid approach when anatomo-functional constraints preclude R0 margins [4,5,6,7,8].

In this context, the choice of spinal instrumentation is pivotal for both biomechanics and oncologic management. Traditional titanium constructs, although reliable, produce MRI/CT artifacts that obscure the surgical bed and adjacent structures. This hinders tumor-bed delineation, delays detection of local recurrence, and complicates dose calculation—especially for charged-particle therapy [9,10,11,12]. Consequently, Carbon-Fiber-Reinforced Polyetheretherketone (CFR-PEEK) implants have emerged as a superior alternative, offering radiolucency for artifact-free imaging and an elastic modulus closer to that of cortical bone, thereby reducing stress shielding and promoting fusion at the bone–implant interface [13,14,15,16,17].

However, a significant technical limitation currently exists: dedicated CFR-PEEK cervical pedicle screws are not commercially available yet. The smallest available screws are designed for the thoracic spine, with a minimum diameter of 4.5 mm. Placing these oversized screws into the smaller and anatomically complex cervical pedicles poses a severe risk of neurovascular injury, particularly to the vertebral arteries and spinal cord [15,18]. Recently, Cofano et al. reported cervical CFR-PEEK pedicle screw fixation using patient-specific 3D-printed template guides for an aggressive vertebral hemangioma, supporting the feasibility of carbon-fiber constructs in cervical oncology [19]. Our report differs by employing robotic navigation to adapt thoracic CFR-PEEK screws to cervical anatomy.

We present the first feasibility report of robot-assisted navigation for the placement of thoracic CFR-PEEK pedicle screws in the cervical spine, describing a workflow utilizing the Globus ExcelsiusGPS^®^ system to address the anatomical mismatch and enable precise screw placement despite challenging vascular anatomy.

## 2. Case Presentation

A 35-year-old male presented with progressive axial neck pain and C7-C8 left radicular pain, refractory to conservative medical management. Neurological examination was unremarkable, with no sensory–motor deficits or signs of myelopathy.

**Figure 1:**
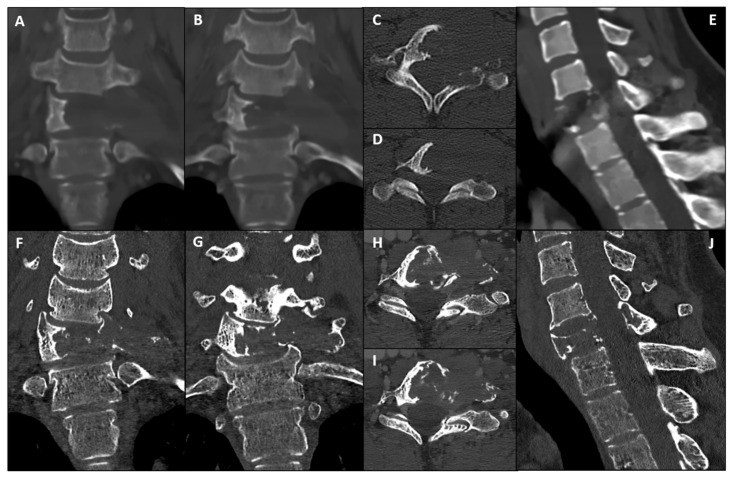
**CT radiographic evolution under Neoadjuvant Denosumab Therapy.** (**A**–**E**) Baseline assessment. Standard bone-window CT reconstructions (Coronal (**A**,**B**), Axial (**C**,**D**), Sagittal (**E**)) at diagnosis, showing a massive, purely osteolytic Giant Cell Tumor involving the C7 vertebral body and posterior elements. Note the voluminous left paravertebral exophytic component, which extends significantly into the soft tissues. (**F**–**J**) Twelve-month follow-up (Photon-Counting CT). Corresponding Coronal (**F**,**G**), Axial (**H**,**I**), and Sagittal (**J**) views acquired via Photon-Counting Detector CT following Denosumab treatment. Comparison: While the images confirm a positive biological response—evidenced by tumor shrinkage and the formation of a hyperdense sclerotic rim (enhanced visualization due to PCCT resolution)—they simultaneously highlight the progressive mechanical failure of the anterior column. Note the development of a severe right-convex cervicothoracic scoliosis (left lateral bending) centered at the C7 fulcrum.

**Figure 2:**
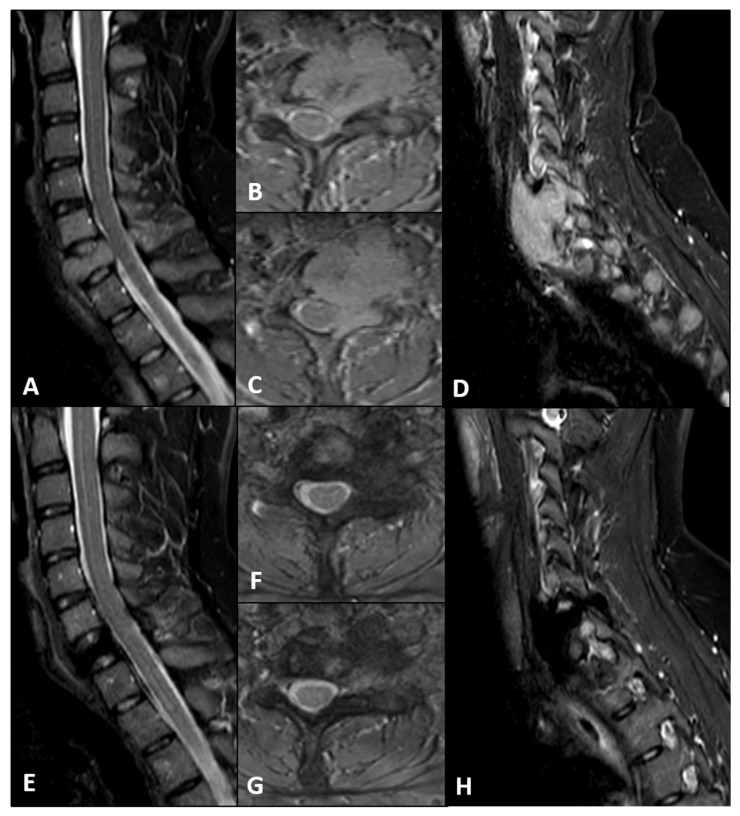
**MRI radiographic evolution under Neoadjuvant Denosumab Therapy.** (**A**–**D**) Baseline assessment. T2-weighted Gradient Echo (GRE) Sagittal (**A**,**D**) and Axial (**B**,**C**) sequences showing a hyperintense, expansile mass involving the C7 vertebra with significant paravertebral extension. (**E**–**H**) Twelve-month follow-up. Corresponding T2-weighted GRE Sagittal (**E**,**H**) and Axial (**F**,**G**) views following Denosumab treatment. Comparison: The follow-up images demonstrate a reduction in tumor volume (shrinkage) and a signal hypointensity consistent with matrix ossification. The sagittal views (**E**,**H**) further document the progression of the local kyphotic deformity.

**Figure 3:**
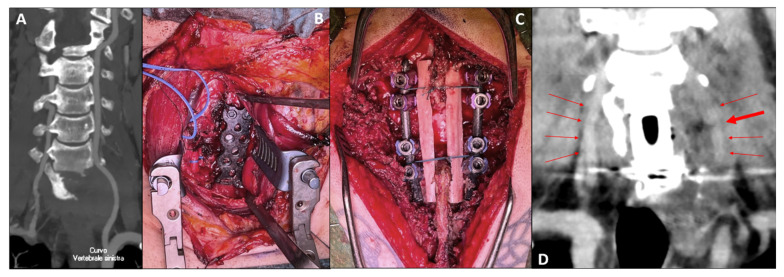
**Vascular assessment and intraoperative reconstruction.** (**A**) Preoperative CT Angiography. Coronal reconstruction demonstrating the critical relationship between the tumor and the left vertebral artery (VA). The vessel appears significantly displaced by the tumor mass. (**B**) Intraoperative view (Anterior Stage). Final construct showing the CFR-PEEK vertebral body replacement cage (KONG® VBR) and anterior cervical plate (BlackArmor®). (**C**) Intraoperative view (Posterior Stage). Final posterior stabilization. The image displays the CFR-PEEK pedicle screws and rods (VADER® system) in position. A double structural fibular allograft is visible, securely tethered to the rods to promote posterior fusion. (**D**) Postoperative CT Angiography. Coronal reconstruction confirming the continuity of both vertebral arteries (red arrows). While the right VA shows normal caliber and enhancement, the repaired left VA remains patent but exhibits reduced contrast density, indicating lower flow velocity. A focal stenosis is clearly identifiable at the anastomotic site (large red arrow), consistent with the vascular repair described.

Computed Tomography (CT) (Figure 1A–E) and Magnetic Resonance Imaging (MRI) (Figure 2A–D) revealed an expansile, osteolytic lesion involving the C7 vertebral body, bilateral pedicles, and the left posterolateral elements. The tumor extended into the left paravertebral soft tissues, laterally displacing the ipsilateral vertebral artery (VA) and abutting its adventitia (270° circumferential involvement) (Figure 3A).

After a multidisciplinary discussion by our institutional Rare Tumor Board, appropriate radiological staging (contrast-enhanced chest and abdominal CT) and a CT-guided trocar biopsy were recommended. No distant spread was detected, and the biopsy confirmed the diagnosis of Giant Cell Tumor of Bone (GCTB).

Based on the multidisciplinary consensus, neoadjuvant therapy with Denosumab (120 mg monthly with loading doses) was initiated to induce tumor ossification and facilitate resection.

Twelve-month follow-up imaging, including CT (Figure 1F–J) and MRI (Figure 2E–H), demonstrated a positive therapeutic response, with mild tumor shrinkage and the formation of a peripheral sclerotic rim. However, the patient developed a de novo cervicothoracic kyphoscoliosis (apex C7) accompanied by worsening mechanical pain. Given the structural instability and symptomatic progression, a two-step surgical strategy was indicated: gross total resection (anterolateral and posterior midline cervical approach) with circumferential carbon-fiber PEEK reconstruction and posterior fusion using structural allografts.

To minimize intraoperative blood loss given the hypervascular nature of the lesion, the patient underwent preoperative Digital Subtraction Angiography (DSA) and selective arterial embolization of the tumor-feeding vessels 24 h prior to surgery.

The surgical procedure was performed as planned. Postoperatively, the patient experienced temporary left vocal cord paralysis (dysphonia) and left-sided Horner’s syndrome, likely related to the anterior cervical dissection and tissue retraction. He was discharged on the ninth postoperative day.

## 3. Surgical Technique

The procedure was performed under continuous intraoperative neurophysiological monitoring (IONM)—somatosensory and motor evoked potentials. A circumferential approach was executed in two sequential steps.

### 3.1. Anterior Step: Corpectomy and VA Repair

With the assistance of an ENT surgeon, an extended left anterolateral cervical approach was performed. The carotid sheath was mobilized laterally and the tracheoesophageal complex medially to expose the prevertebral space. Following bilateral elevation of the longus colli muscles, the anterior aspect of C7, distorted by the tumor, was fully visualized.

Discectomies were performed at C6-C7 and C7-T1. Drilling of the reactive sclerotic rim allowed access to the tumor core, which exhibited a fibrous consistency. Systematic intralesional curettage was carried out, completing the C7 corpectomy and extending to the left paravertebral compartment.

During the meticulous dissection of the left vertebral artery (VA)—which was highly adherent to and displaced by the tumor mass—through a retro-jugular corridor, an iatrogenic injury to the vessel occurred. The vascular surgery team performed an immediate repair via end-to-end anastomosis. Patency and pulsatility were confirmed intraoperatively using a Doppler probe. Throughout this event, hemodynamic parameters remained stable, and IONM showed no sustained deviations from baseline during the remainder of the procedure.

Following tumor removal, anterior column reconstruction (ACR) was achieved using a radiolucent CFR-PEEK vertebral body replacement cage (KONG^®^ VBR system, Icotec AG, Altstätten, Switzerland). The construct was secured with a CFR-PEEK anterior cervical plate (BlackArmor^®^ Anterior Cervical Plate system, Icotec AG, Altstätten, Switzerland) to ensure immediate stability (Figure 3B). An operating microscope was utilized throughout the procedure for enhanced visualization.

### 3.2. Posterior Step: Robot-Assisted Instrumentation

The patient was repositioned prone. A posterior midline cervical approach enabled the exposure of levels from C5 to T2. Using microsurgical technique, residual tumor infiltration of the posterolateral elements was excised, ensuring the preservation of nerve roots and the spinal cord.

**Figure 4:**
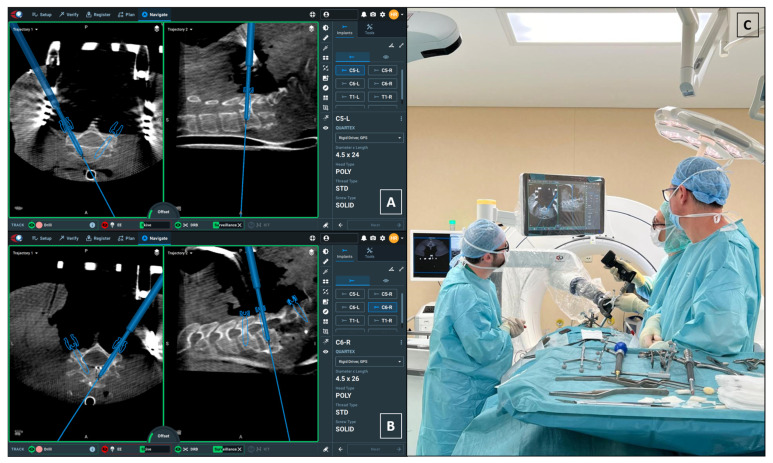
**Intraoperative robotic planning and surgical setup.** (**A**,**B**) Interface screenshots of the ExcelsiusGPS® navigation system during trajectory planning for the left C5 (**A**) and right C6 (**B**) pedicle screws. Each panel displays the corresponding axial (left sub-panel) and sagittal (right sub-panel) projections. The axial views clearly demonstrate the strategic planning: while the left side allows for a standard trajectory, the right-side trajectories (visible in panel (**B**) and as the contralateral plan in panel (**A**)) show the intentional, small, controlled violation of the medial pedicle cortex (medial breach). This strategy was employed to accommodate the 4.5 mm screw diameter while strictly avoiding the lateral vertebral artery canal. (**C**) Intraoperative photograph showing the surgical field, the robotic arm, and the O-Arm™ imaging system. Crucially, the registration CT scan was acquired with the cervical self-retaining retractors already secured in place; this technical nuance prevents vertebral realignment or shift occurring after imaging, ensuring that the navigation data perfectly matches the real-time anatomy during screw placement. In these navigation views, orientation is ipsilateral (right displayed on the right, left on the left).

Following complete skeletal exposure and the positioning of self-retaining retractors, an intraoperative Cone-Beam CT (CBCT) scan for robotic registration was acquired using the O-Arm™ Surgical Imaging System (Medtronic, Minneapolis, MN, USA). The image dataset was automatically registered to the robotic system to facilitate the subsequent planning phase. Registration accuracy was verified intraoperatively according to the standard ExcelsiusGPS^®^ workflow. The registration CBCT was acquired with the cervical self-retaining retractors already secured in place in order to minimize the risk of vertebral shift after imaging and to ensure real-time correspondence between the navigation dataset and the surgical anatomy. Throughout trajectory planning and screw placement, the skive, DRB, and surveillance indicators remained within the acceptable range (green status on the navigation interface), confirming stable tracking and absence of intraoperative displacement (Figure 4).

Posterior stabilization was planned from C5 to T2 using the VADER^®^ pedicle screw system (Icotec AG). A critical technical challenge was the bilateral mismatch between implant dimensions and patient anatomy: the smallest available CFR-PEEK screws have a diameter of 4.5 mm, whereas the cervical pedicles at C5 and C6 were significantly narrower on both sides. Specifically, the left pedicles measured 3.7–4.0 mm, while the right pedicles were even smaller, ranging from 3.2 to 3.7 mm (Table 1).

This resulted in a relevant screw–pedicle mismatch, with a minimum pedicle width of 3.2 mm compared with a 4.5 mm screw diameter. Given the 3.2–3.7 mm right pedicle diameter and the requirement to preserve the only intact vertebral artery, any lateral deviation would have risked violation of the vertebral artery canal; therefore, a minimal planned medial cortical encroachment represented the safest geometrical corridor. On the right side, a minimal and intentionally planned medial cortical encroachment was selected as the safest trajectory. This was defined on the basis of preoperative CT-based anatomical assessment and intraoperative three-dimensional navigation and subsequently verified with a final intraoperative CBCT.

While the left side presented a more physiological trajectory, the situation on the right was critical. Given the prior injury to the left VA during the anterior stage, preserving the right vertebral artery became mandatory. This necessitated placing an oversized screw into a hypoplastic pedicle with zero margin for lateral error.

Given the inherent risks, robotic navigation (ExcelsiusGPS^®^, Globus Medical, Audubon, PA, USA) was employed for screw trajectory planning and placement (Figure 4).

- Left Side (Repaired VA): The larger pedicle diameter allowed for a standard transpedicular trajectory.
- Right Side (Intact VA): Given the small pedicle diameter and the need to minimize lateral breach (risking injury to the only patent VA), we planned a small, controlled medial cortical violation, with limited encroachment into the canal, facilitated by robotic navigation. This choice is consistent with anatomical/clinical observations indicating higher vertebral artery risk with lateral breaches and short-term tolerance of limited medial violations [20,21,22].

All screws (C5–C6, T1–T2) were thoracic CFR-PEEK pedicle screws (4.5 × 25 mm) and were placed successfully using the robotic arm. Screw position was subsequently assessed on early postoperative CT using a standardized pedicle breach grading system (Gertzbein–Robbins classification) [20], reported per level and side (Table 2). Intraoperative verification, performed via a final intraoperative CBCT scan, confirmed the planned trajectories with no neurovascular compromise. Pre-contoured CFR-PEEK rods were secured to complete the construct.

**Figure 5:**
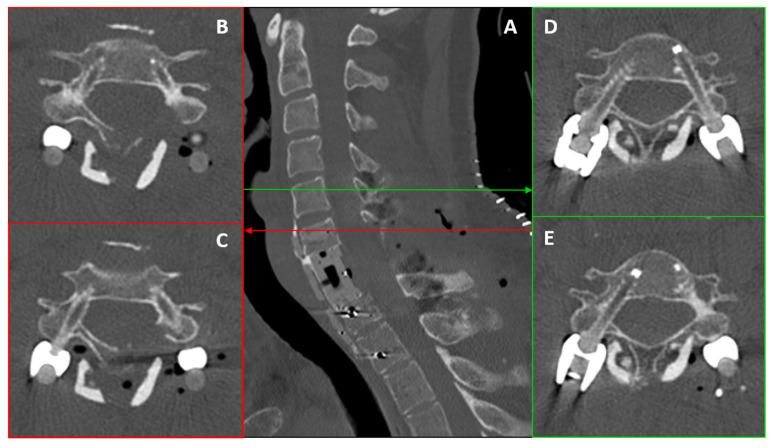
**Postoperative Computed Tomography (CT) assessment.** (**A**) Central midsagittal reconstruction showing anterior column restoration. Note the radiolucency of the CFR-PEEK cage and anterior plate, identified by radiopaque markers, allowing artifact-minimizing visualization of the canal and fusion bed. (**B**,**C**) Axial views on the left side of the panel, corresponding to C6. (**D**,**E**) Axial views on the right side of the panel, corresponding to C5. In these postoperative CT images, orientation follows standard radiologic convention (patient’s right displayed on the left) and is therefore mirrored relative to the ipsilateral orientation used in Figure 4. The axial images confirm the planned trajectories: left-sided screws follow a standard path, whereas right-sided screws at both levels (particularly evident at C5 in (**D**,**E**), also appreciable at C6 in (**B**,**C**)) exhibit the intentional, small, controlled medial cortical breach. This trajectory accommodated the 4.5 mm screw within hypoplastic pedicles, preserving the lateral wall and protecting the right vertebral artery. For clarity, the corresponding intraoperative planning images for the same slices are shown in Figure 4A,B and are superimposable on the postoperative CT; screw contours, pedicle cortices, and the relationship to the vertebral artery are indicated for direct planned-versus-achieved comparison.

To promote posterior fusion, a double structural fibular allograft was positioned along the spinolaminar surfaces and securely tethered to the rods to minimize displacement (Figure 3C). Final inspection confirmed adequate decompression and rigid circumferential stabilization. The posterior stage was also performed under microscope magnification. Early postoperative CT imaging subsequently confirmed the accurate placement of the instrumentation, strictly consistent with the specific robotic planning, with preservation of the lateral pedicle wall at all instrumented levels (Figure 5). On the same postoperative imaging, angiographic sequences demonstrated the patency of the repaired left vertebral artery, albeit with reduced contrast enhancement indicating lower flow and a focal stenosis at the anastomotic site (Figure 3D).

**Figure 6:**
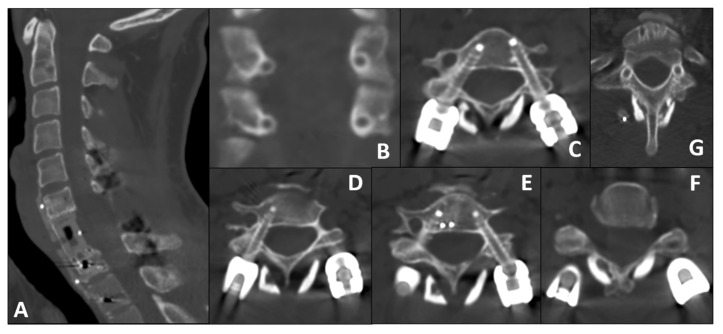
**Three-month postoperative CT scan (bone window) confirming stable circumferential reconstruction** (**A**) Midline sagittal view showing adequate press-fit of the anterior cage without subsidence. (**B**) Coronal reconstruction at C5–C6 demonstrating the planned medial trajectory of the right-sided pedicle screws, unchanged compared with the immediate postoperative CT and without signs of loosening. (**C**) Axial view at C5 confirming correct screw positioning and construct stability. (**D**,**E**) Axial views showing the right and left pedicle screws, respectively, with no evidence of mobilization; the medial cortical encroachment on the right side is unchanged. (**F**,**G**) Axial views at the cranial and caudal ends of the posterior construct demonstrating early osseous integration of the structural bone graft. No radiological evidence of residual or recurrent disease is observed.

At three-month clinical and radiological evaluation, the stability and accuracy of the previously described reconstruction were confirmed. The patient was neurologically intact, with complete resolution of dysphonia and Horner’s syndrome. CT imaging demonstrated complete tumor removal, correct positioning of the instrumentation, and early posterior fusion (Figure 6). Duplex ultrasonography performed by the vascular surgery team showed persistent patency of the repaired vertebral artery, with reduced but preserved flow compared with the contralateral side.

## 4. Discussion

The surgical management of primary spinal tumors requires a delicate balance between oncological control and functional preservation. While En-Bloc resection remains the gold standard for reducing recurrence, anatomical constraints at the cervicothoracic junction often necessitate a deliberate intralesional piecemeal resection combined with robust stabilization [4,5,21].

In this context, our case highlights the integration of CFR-PEEK instrumentation and robotic navigation to overcome specific hardware limitations and anatomical risks.

### 4.1. CFR-PEEK Instrumentation: Potential Advantages

Titanium implants, traditionally the standard for stabilization, generate significant susceptibility artifacts on MRI and CT. In oncological follow-up, these artifacts can obscure the surgical bed, delaying the detection of local recurrence—a critical issue in GCTB which has a high recurrence rate. CFR-PEEK implants are radiolucent and reduce imaging artifacts, allowing for precise postoperative surveillance and accurate dose planning for adjuvant charged-particle therapy (proton/carbon ion) [13,14,15,16,17,22]. Furthermore, the elastic modulus of CFR-PEEK closely resembles that of cortical bone, which may reduce stress shielding and promote better fusion at the bone–implant interface [16,23].

### 4.2. Robotic Planning Under Hardware Constraints: Approach, Safety, and Alternatives

A major limitation of current CFR-PEEK systems is the lack of cervical-specific instrumentation; the smallest screws are thoracic (minimum diameter 4.5 mm). In cervical pedicles, these dimensions increase the risk of lateral cortical breach and vertebral artery injury [21,24,25]. In this case, robotic guidance (ExcelsiusGPS^®^) facilitated submillimetric trajectory planning [26,27]. On the right side (intact VA), we planned a small, controlled medial cortical violation to reduce VA risk, consistent with anatomical/clinical observations of higher VA risk with lateral breaches and short-term tolerance of limited medial violations [28,29]. This approach protected the only patent VA while maintaining rigid fixation. Adapting thoracic hardware to cervical anatomy with robotic precision is the primary technical contribution of this report.

In this young oncologic patient with C7 involvement and circumferential reconstruction, rigid posterior fixation with pedicle purchase was prioritized to span the cervicothoracic junction with a longer lever arm and higher pullout strength [30], supporting alignment and enabling precise, artifact-minimizing surveillance and particle-therapy planning [10,11,12,13,14,31].

In the present case, the indication for pedicle fixation was primarily driven by oncological and reconstructive requirements rather than by a purely technical preference. Following C7 corpectomy and circumferential reconstruction across the cervicothoracic junction, a long posterior construct was required to provide a sufficient lever arm across the resected segment, maximize pullout strength, and maintain long-term alignment in a young patient. In addition, the use of a fully radiolucent construct was considered essential to enable artifact-minimizing postoperative surveillance and accurate radiotherapy planning. Alternative strategies, including lateral mass screw fixation, were carefully evaluated; however, their lower pullout strength and shorter moment arm across a resected segment were judged insufficient for the intended oncological pathway [4,6,19,24]. Pediculectomy would have further reduced the available cortical constraints for a 4.5 mm screw. This robotic workflow complements template-guided techniques described by Cofano et al. [19], offering an alternative when patient-specific guides are unavailable or when intraoperative plan adjustment is required.

### 4.3. Multimodal and Complication Management

The complexity of this case was further compounded by the vascular anatomy. The preoperative use of Denosumab was instrumental in defining the tumor margins and increasing tumor consistency, but with a potential risk of fibrotic adhesions to the surrounding tissues. Minimally invasive or intralesional treatment strategies, including local adjuvant therapies such as steroid injections, have been proposed for selected giant cell tumors, particularly in anatomically expendable sites or in the absence of structural instability [32,33,34]. However, in the mobile spine these approaches may be insufficient when the lesion causes vertebral body destruction, progressive deformity, or circumferential involvement of critical neurovascular structures. In the present case, the development of a cervicothoracic kyphoscoliotic deformity and the need for circumferential reconstruction represented the main factors leading to an extensive surgical strategy. The intraoperative injury to the left VA underscores the hazards of dissecting tumors encasing vascular structures [28,29]. However, the successful immediate repair and the subsequent robotic planning—which treated the left side as “standard” and the right side as “critical”—showcase the importance of adaptability in complex spine surgery.

### 4.4. Limitations, Feasibility, and Applicability

This single-case report focuses on technical feasibility rather than longitudinal outcomes. Evidence directly comparing medial versus lateral breach is limited; anatomical and observational data suggest a greater vertebral artery risk with lateral breaches and a short-term clinical tolerance of small, controlled medial violations [20,24]. Long-term effects (e.g., epidural fibrosis, chronic cord abrasion, or delayed neurological deterioration) remain uncertain. Reproducibility may be limited to experienced centers with robotic navigation and extensive familiarity with cervical pedicle techniques [23,28,29]. Follow-up is currently limited to three months; therefore, long-term durability of fixation, solid fusion, local control, and delayed neurological effects cannot be assessed and were not primary objectives of this report. Although no immediate neurological or vascular complications were observed, the long-term tolerance of a planned medial cortical encroachment in the cervical spine remains unknown, and delayed neurological or vascular changes cannot be excluded. For this reason, this strategy should be considered only in highly selected cases and in centers with extensive experience in cervical pedicle instrumentation and robotic navigation. Furthermore, the adoption of robotic assistance requires dedicated infrastructure, intraoperative three-dimensional imaging, and a specifically trained surgical team, with consequent cost implications, which may further limit the reproducibility of this workflow outside high-volume referral centers.

## 5. Conclusions

To our knowledge, this is the first feasibility report of robot-assisted placement of thoracic CFR-PEEK pedicle screws in the cervical spine. In carefully selected oncologic cases requiring artifact-minimizing imaging where cervical-specific carbon-fiber implants are not yet available, this approach may be considered in experienced centers with access to robotic navigation. The technique included a planned, small medial cortical violation to mitigate vertebral artery risk; while short-term tolerance is observed, long-term safety remains uncertain. At three-month follow-up, clinical and radiological evaluation confirmed neurological integrity, correct screw positioning, and early posterior fusion, supporting the technical feasibility of this strategy. This experience adds to emerging reports on cervical CFR-PEEK fixation, including template-guided approaches [19], by demonstrating a robot-assisted workflow in a different pathology and hardware setting. Long-term follow-up is required to assess oncologic control, screw stability, delayed neurological complications, and vertebral artery status.

## Figures and Tables

**Table 1:** **Cervical pedicle width and screw–pedicle mismatch.** Quantitative relationship between cervical pedicle width and the minimum available CFR-PEEK screw diameter, showing the planned dimensional mismatch.

Level	Side	Pedicle Width (mm)	Screw Diameter (mm)	Mismatch (mm)
C5	Right	3.2–3.7	4.5	+0.8–1.3
C5	Left	3.7–4.0	4.5	+0.5–0.8
C6	Right	3.2–3.7	4.5	+0.8–1.3
C6	Left	3.7–4.0	4.5	+0.5–0.8

**Table 2:** **Early postoperative CT-based per-screw accuracy assessment** **(Gertzbein–Robbins classification).**

Level	Side	Intended Medial Encroachment	GR Grade	Revision Required
C5	Right	Yes	C	No
C5	Left	No	A	No
C6	Right	Yes	C	No
C6	Left	No	A	No
T1	Right	No	A	No
T1	Left	No	A	No
T2	Right	No	A	No
T2	Left	No	A	No

## Data Availability

The original contributions presented in this study are included in the article. Further inquiries can be directed to the corresponding author.
